# Letter to the editor regarding “Kılıçarslan A, Kurt Çevik G, Doğan M, Aksoy Altınboğa A, Ceran F, et al. Does CD47 expression have prognostic significance in classical Hodgkin lymphoma? Turkish Journal of Medical Sciences: 2025; 55 (1): 22”

**DOI:** 10.55730/1300-0144.6056

**Published:** 2025-05-04

**Authors:** Burcu BABA, Ayşegül AKBAY

**Affiliations:** Department of Medical Biochemistry, Faculty of Medicine, Yüksek İhtisas University, Ankara, Turkiye

The article by Kılıçarslan et al., titled “Does CD47 expression have prognostic significance in classical Hodgkin lymphoma?”, is highly interesting [1]. We are writing to highlight recent advances in identifying biochemical markers for classical Hodgkin lymphoma (cHL) and their potential correlation with CD47 as a diagnostic and prognostic tool. Considering the difficulties in early diagnosis and disease monitoring, there is a growing need for reliable biomarkers to complement histopathological examinations and imaging techniques.

Several promising biochemical markers associated with cHL pathogenesis have been proposed in recent studies. Among these markers, soluble CD30 (sCD30) and soluble CD163 (sCD163), which are suggested to reflect tumor burden and inflammatory activity, have been found [2]. Increased levels of serum thymus and activation-regulated chemokine (TARC/CCL17) have attracted attention as a highly sensitive marker, correlating with disease activity and treatment response [3,4].

One particularly notable finding was that the immune checkpoint protein CD47, known as the “don’t eat me” signal, allows malignant Reed-Sternberg cells to escape phagocytosis. Recent research suggests that increased CD47 expression in cHL may be associated with disease aggressiveness and immune evasion. Therefore, CD47 may be considered a potential target for immunotherapy. Furthermore, emerging data indicate that serum levels of CD47 and its interaction with macrophage markers such as sCD163 could serve as valuable indicators for prognosis and disease monitoring [5].

Combining traditional biochemical markers with CD47-related pathways may enhance diagnostic accuracy and provide new insights into cHL progression. However, further large-scale studies are needed to validate these findings and determine their clinical applicability.

Considering the significance of these advancements, we propose that Turkish Journal of Medical Sciences consider publishing a focused review or special issue on the evolving role of biochemical markers, including CD47, in cHL diagnosis and follow-up. This would facilitate discussion on integrating these biomarkers into clinical practice for improved patient outcomes.

Thank you for your time and consideration. We look forward to your response and to further contributions in this promising area of research.

## References

[1] Kılıçarslan A Kurt Çevik G Doğan M Aksoy Altınboğa A Ceran F Does CD47 expression have prognostic significance in classical Hodgkin lymphoma? Turkish Journal of Medical Sciences 2025 55 1 22 10.55730/1300-0144.5958 PMC11913508 40104289

[2] Plattel WJ Alsada ZN van Imhoff GW Diepstra A van den Berg A Biomarkers for evaluation of treatment response in classical Hodgkin lymphoma: comparison of sGalectin-1, sCD163 and sCD30 with TARC British Journal of Haematology 2016 175 5 868 875 10.1111/bjh.14317 27610595

[3] Hsi ED Li H Nixon AB Schöder H Bartlett NL Serum levels of TARC, MDC, IL-10, and soluble CD163 in Hodgkin lymphoma: a SWOG S0816 correlative study Blood 2019 133 16 1762 1765 10.1182/blood-2018-08-870915 30723079 PMC6473498

[4] Zijtregtop EAM van der Strate I Beishuizen A Zwaan CM Scheijde-Vermeulen MA Biology and Clinical Applicability of Plasma Thymus and Activation-Regulated Chemokine (TARC) in Classical Hodgkin Lymphoma Cancers (Basel) 2021 13 4 884 10.3390/cancers13040884 33672548 PMC7923750

[5] Gholiha AR Hollander P Löf L Glimelius I Hedstrom G Checkpoint CD47 expression in classical Hodgkin lymphoma British Journal of Haematology 2022 197 5 580 589 10.1111/bjh.18137 35301709 PMC9310712

